# Light-dependent processes in algae across scales: from cellular physiology to ecological organization

**DOI:** 10.1093/plphys/kiag524

**Published:** 2026-07-24

**Authors:** Armin Hallmann

**Affiliations:** Department of Cellular and Developmental Biology of Plants, University of Bielefeld, Universitätsstr. 25, Bielefeld D-33615, Germany

## Abstract

Sunlight provides both energy for photosynthesis and environmental information for algae. Beyond supporting a large share of global primary production, light encodes cues that regulate physiology, development, and ecological performance. This review examines how algae detect and interpret light and how these signals are integrated into cellular and organismal responses. Across photosynthetic algal lineages, many light responses arise from the integration of 2 input streams: photoreceptor-mediated sensory signaling and metabolic redox signals generated by photosynthetic electron transport. Across algal lineages, diverse photoreceptors—including rhodopsins, cryptochromes, phototropins, phytochromes, UV-B photoreceptor UVR8, and lineage-specific sensors such as aureochromes—detect spectral, directional, and temporal properties of light. Their outputs converge with chloroplast-derived signals—including redox state, reactive oxygen species, ion fluxes, and second messengers—to form recurring regulatory architectures that coordinate photosynthesis, photoprotection, pigment biosynthesis, metabolism, and gene expression. Through these pathways, light also regulates development and behavior, including motility, phototropism, circadian rhythms, cell-cycle progression, and life-cycle transitions. These processes operate across biological scales, from intracellular signaling to ecological organization, enabling algae to occupy diverse light environments. Emerging genomic, structural, and functional approaches will clarify how photoreceptor diversity and metabolic sensing are integrated across scales and may support predictive models linking cellular regulation to ecological performance under changing light climates.

## Introduction

Light is a central organizing factor in algal biology. It sustains photosynthetic growth, structures aquatic light environments, and provides information that regulates physiology, development, behavior, and ecological performance. Because algae span multiple evolutionary lineages, cellular organizations, and life histories, light-dependent processes cannot be understood as a single uniform program. Instead, they are best viewed as a set of recurring regulatory problems—how to capture light, avoid excess excitation, interpret spectral and temporal information, and translate these inputs into cellular and ecological responses—that have been solved in different ways across algal diversity.

### Algae and their diversity

In this review, the term “algae” is used in a functional rather than strictly phylogenetic sense to refer to photosynthetic eukaryotes outside land plants. This includes lineages with primary plastids, such as green and red algae, as well as groups with secondary or tertiary plastids, including stramenopiles, haptophytes, cryptophytes, euglenophytes, and dinoflagellates. These organisms differ widely in evolutionary origin, cellular organization, life history, habitat, and motility, encompassing coccoid, filamentous, and flagellated unicells, benthic and pelagic lifestyles, colonial and multicellular forms, and more complex macroalgae (Figure S1). Consequently, the examples discussed below are not intended to imply uniform mechanisms across all algae. Instead, they are used comparatively to distinguish recurring regulatory principles from lineage- or lifestyle-specific implementations.

### Light as coupled energetic and informational input

**Figure 1:**
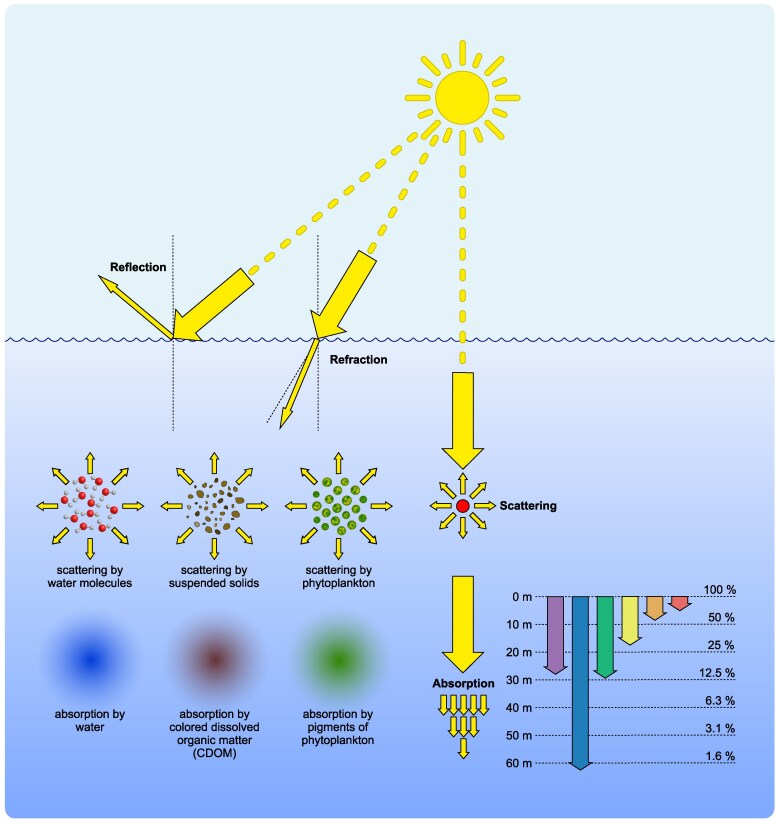
Optical processes shaping the underwater light field. Incident solar radiation is partially reflected at the air–water interface, while the refracted component enters the water column where it is progressively modified by wavelength-dependent scattering (Rayleigh and Mie processes) and absorption by pure water, CDOM, and algal pigments. These combined processes determine the vertical decline of light intensity and spectral diversity in natural waters and establish depth-dependent optical niches for photosynthesis and photoreception. Conceptual illustration synthesized from information presented in Mobley (1994); Falkowski and Raven (2007); Kirk (2010); Contreras-Silva et al. (2012); Larkum et al. (2018); Kim and Lee (2022); Huang et al. (2024); Xu et al. (2025a).

Sunlight drives algal photosynthesis and growth. It sustains aquatic ecosystems responsible for approximately 45% to 50% of global primary production (Field et al. 1998; Falkowski and Raven 2007; Raven 2009; Falkowski 2012). Across photosynthetic algae, however, light functions not only as an energy source but also as an environmental signal. Its intensity, spectral composition, direction, and temporal structure encode cues that regulate physiology, development, and ecological performance (Mobley 1994; Morel and Maritorena 2001; Falkowski and Raven 2007; Kirk 2010; Larkum et al. 2018; Hintz et al. 2022) (Fig. 1). Through these processes, different algal lineages occupy diverse aquatic and subaerial light environments. Mechanistically, many algal light responses can be understood as arising from the integration of 2 interacting input streams:

1. photoreceptor-mediated sensory input, and
2. metabolic redox input generated by photosynthetic electron transport.

Rather than cataloging photoreceptors, this review focuses on how light perception produces physiological and ecological responses, with emphasis on its coupling to redox state, carbon status, second messengers (Ca^2+^, cAMP/cGMP), and retrograde signaling. Addressing this requires moving beyond descriptive inventories and pathway-level studies toward models that connect fluctuating light environments to cellular decisions, organismal traits, and ecological patterns. Progress remains constrained by an uneven evidence base: Mechanistic insight is deep in a few model systems, whereas phylogenetic breadth, quantitative comparability, and causal links to ecology remain limited.

Diverse algal photoreceptors often converge with metabolic and redox signals on shared intracellular signaling networks (Hegemann 2008; Duanmu et al. 2017; Rockwell and Lagarias 2020; Petersen et al. 2021; Petersen et al. 2022; Hallmann 2025). In many unicellular algae, photosynthesis, growth, cell-cycle progression, motility, and life-cycle transitions occur within a single cellular context with little tissue buffering, allowing light fluctuations to propagate rapidly from photochemistry to gene expression, behavior, and developmental state. Building on these established observations, this review uses 4 recurring functional themes as an organizational framework rather than proposing that they represent uniform mechanisms across algal diversity:

1. **Energetic–sensory coupling**: Redox changes in the photosynthetic electron transport chain act as regulatory signals, including plastoquinone redox state and lumen acidification (Minagawa 2011; Petroutsos et al. 2016; Roach et al. 2020).
2. **Spectral specialization layered onto metabolic control**: Photoreceptors refine responses to wavelength, photoperiod, and directionality (Hegemann 2008; Duanmu et al. 2017; Rockwell and Lagarias 2020; Petersen et al. 2021; Hallmann 2025).
3. **Multiscale integration**: Molecular signaling cascades scale to physiological acclimation, development, and ecological performance (Petroutsos et al. 2016; Mittler 2017; Sasso et al. 2018; Petersen et al. 2021; Vierock and Hegemann 2023).
4. **Feedback-dominated regulation**: Ecological light climate feeds back onto cellular metabolism, which in turn reshapes light perception and signaling capacity (Falkowski and Raven 2007; Kirk 2010; Petroutsos et al. 2016; Duanmu et al. 2017; Roach et al. 2020).

**Figure 2:**
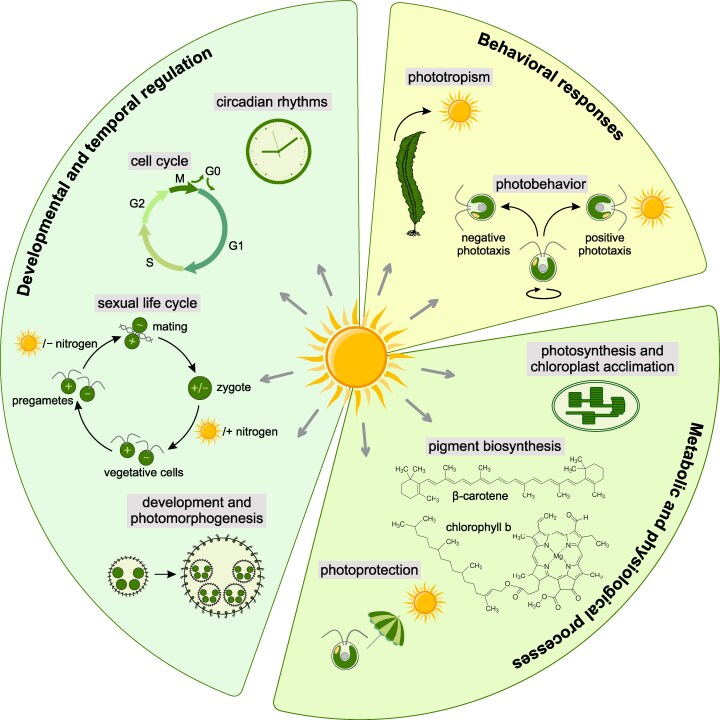
Light-dependent physiological, behavioral, and developmental processes in algae. Conceptual overview of major light-regulated processes documented across algal systems, organized into 3 functional domains: metabolic and physiological processes, behavioral responses, and developmental and temporal regulation. These include photosynthesis and chloroplast acclimation, photoprotection, pigment biosynthesis, photobehavior, including phototaxis and phototropism, circadian regulation, control of the cell cycle, developmental programs and photomorphogenesis, and transitions of the sexual life cycle. Arrows radiating from the central light source illustrate how light acts as a common regulatory input across these processes. Redrawn and modified after Petersen et al. (2021), with additional elements added.

These principles define recurring functional themes that are implemented differently depending on lineage, lifestyle, habitat, and cellular organization. They provide the conceptual scaffold for the review and are linked to major physiological, developmental, and behavioral processes summarized in Fig. 2.

### Energetic–sensory coupling of light

**Figure 3:**
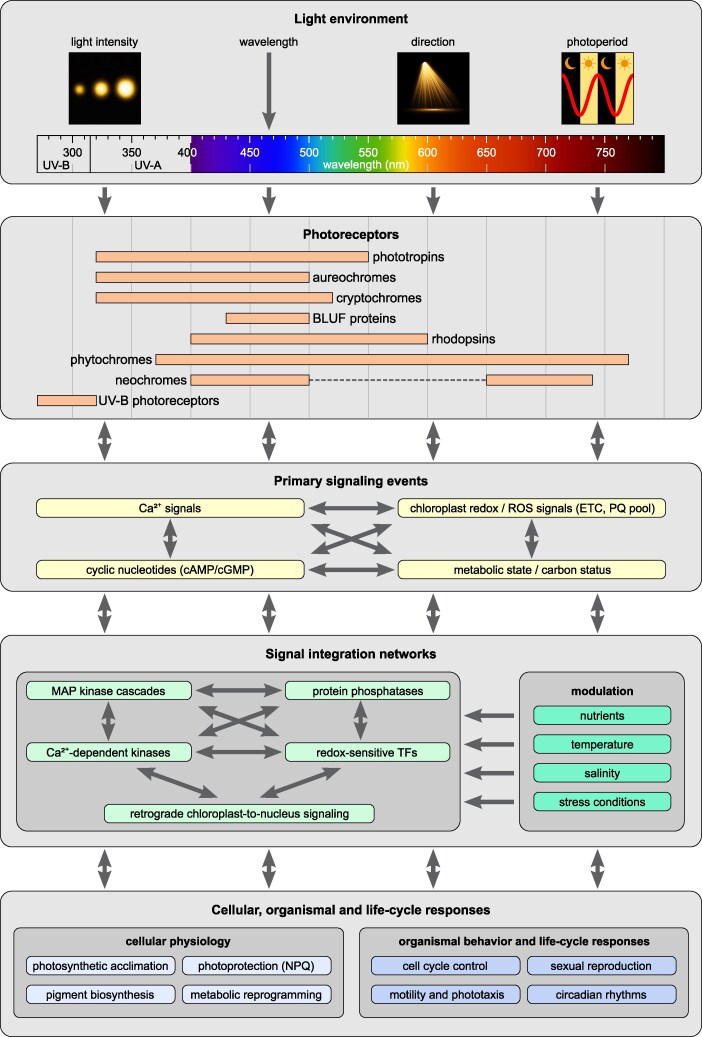
Architecture of light-triggered signaling networks in algae. Schematic overview illustrating how diverse algal photoreceptors can be integrated into recurring signaling networks to generate coordinated physiological, developmental, and behavioral responses. Distinct light parameters—including intensity, spectral quality, direction, and photoperiod—are perceived by multiple classes of photoreceptors spanning UV-B to far-red wavelengths (approximate spectral sensitivities are indicated). Photoreceptor activation initiates primary intracellular signaling events. Early signal transduction involves second messengers and metabolic signals, including transient Ca^2+^ signals, ROS, cNMPs (cAMP/cGMP), chloroplast redox signals linked to photosynthetic electron transport, and changes in metabolic or carbon status. These signals converge in interconnected regulatory networks comprising MAP kinase cascades, calcium-dependent protein kinases, protein phosphatases, redox-sensitive transcription factors, and chloroplast-to-nucleus retrograde signaling pathways. Signal integration enables context-dependent modulation by additional environmental factors, including nutrient availability, temperature, salinity, and stress conditions. Downstream effector processes include photosynthetic acclimation, photoprotection, pigment biosynthesis, metabolic reprogramming, regulation of the cell-cycle and life-cycle transitions, motility and phototaxis, and circadian rhythms. Together, this architecture illustrates that many algal light responses arise from dynamic and modular signaling networks rather than linear pathways, providing a mechanistic basis for the pronounced photophysiological plasticity observed across algal lineages and habitats. Bidirectional arrows indicate reciprocal feedback regulation between signaling layers. In several algal systems, photoreceptor abundance, activity, and stability are additionally modulated by intracellular signaling states, including Ca^2+^ signaling, cNMPs, chloroplast redox poise, metabolic status, and acclimation state (Trippens et al. 2012; König et al. 2017; Böhm et al. 2019; Jaubert et al. 2022; Vierock and Hegemann 2023; Wolfram et al. 2023, 2024; Im et al. 2024b). The conceptual framework summarized in this figure was synthesized from information presented in the literature cited throughout this review.

In photosynthetic algae, the energetic and informational roles of light form a continuum, not 2 discrete functions. In aquatic environments, wavelength-dependent scattering and absorption generate depth-dependent gradients in intensity and spectral composition that structure ecological light niches (Fig. 1) (Mobley 1994; Pope and Fry 1997; Morel and Maritorena 2001; Kirk 2010; Hintz et al. 2022; Bi et al. 2023). Light-driven electron transport therefore acts as both an energy source and a regulatory signal (Fig. 3) (Minagawa 2011; Roach et al. 2020).

State transitions provide a clear example of energetic–sensory coupling. In *Chlamydomonas reinhardtii*, the thylakoid kinase STT7 senses the redox state of the plastoquinone pool and phosphorylates light-harvesting complex (LHC) II, redistributing excitation energy between photosystem II (PSII) and photosystem I (PSI) (Lemeille and Rochaix 2010; Ünlü et al. 2014; Minagawa and Tokutsu 2015). Related excitation-balancing responses also occur in phycobilisome-containing algae, including red algae, but are implemented through different antenna architectures. In these systems, adjustment of phycobilisome coupling to PSII and PSI, including spillover-like energy redistribution, can rebalance photosystem excitation without relying on the LHC II-phosphorylation mechanism characteristic of green algae (Biggins and Bruce 1989; Delphin et al. 1996; Yokono et al. 2011). In both cases, the redox consequences of light absorption directly reorganize the photosynthetic apparatus, without requiring a dedicated upstream photoreceptor. Thus, lineage-specific mechanisms can converge on the same physiological outcome: balancing excitation pressure between PSII and PSI under changing light conditions.

A related example is nonphotochemical quenching (NPQ), which dissipates excess excitation energy as heat. In chlorophytes, LHC stress-related (LHCSR) proteins respond to lumen acidification, which reflects imbalances between light harvesting and carbon fixation (Peers et al. 2009; Bonente et al. 2011; Allorent et al. 2013; Niyogi and Truong 2013). Other algal lineages use different molecular implementations of the same photoprotective logic, including LHCX-dependent quenching in diatoms and mechanistically distinct, often phycobilisome-associated quenching responses in red-lineage systems (Bailleul et al. 2010; Goss and Lepetit 2015; Taddei et al. 2016, 2018). Although NPQ acts as a physiological effector, its regulation integrates photoreceptor input with carbon status and retrograde signals (Petroutsos et al. 2016; Roach et al. 2020).

Beyond photochemistry-derived redox signals, photoreceptors also modulate metabolism and development. Blue-light perception via phototropin regulates starch accumulation in *Volvox carteri* and *C. reinhardtii*, linking wavelength detection to carbon partitioning (Yuan et al. 2025). Photon flux sets energetic capacity, whereas spectral quality reshapes allocation. Photoacclimation thus emerges from interactions among redox signals, photoreceptor inputs, and metabolic state (Fig. 3).

## Spectral specialization and receptor architecture in algae

### Modular receptor design

Across algal lineages, light-sensing repertoires span UV-B to far-red, with overlapping absorption bands across receptor classes (Fig. 3) (Lariguet and Dunand 2005; Rockwell et al. 2014; Duanmu et al. 2017; Rockwell and Lagarias 2020; Shankar et al. 2022; Hallmann 2025). Together with lineage-specific light-harvesting pigments, whose absorption properties are summarized in Figure S2, this combined photoreceptor–pigment architecture shapes how algal cells sense and use light intensity, spectral composition, direction, and temporal structure (Hegemann 2008; Duanmu et al. 2017; Larkum et al. 2018; Petersen et al. 2021; Hallmann 2025).

Many algal photoreceptors act as modular spectral sensors that feed into common signaling pathways. However, functional characterization still lags behind sequence-based discovery, and for many receptors downstream wiring remains incompletely resolved; thus, apparent convergence may reflect convergent outputs rather than homologous signaling architecture. Table 1 summarizes major photoreceptor families, while the main text focuses on functional classes: (i) flavin-based blue/UV-A sensors, (ii) retinal-based rhodopsins, (iii) bilin-based phytochromes and related systems, (iv) hybrid receptors, and (v) UV-B-specific UVR8, which uses an intrinsic tryptophan chromophore rather than an external prosthetic group (Hallmann 2025).

**Table 1:** Major classes of algal photoreceptors and their integration into intracellular signaling networks.

Photoreceptor class	Principal spectral range	Major downstream integration nodes	Dominant functional outputs	Representative algal lineages
Phototropins (LOV kinases)	Blue	Ca^2+^ transients, kinase cascades	Phototaxis, chloroplast movement, photoprotection (NPQ regulation)	Chlorophyta
Aureochromes (LOV–bZIP transcription factors)	Blue	Direct transcriptional control	Direct transcriptional regulation	Stramenopiles
Cryptochromes	Blue/UV-A	Redox modulation, transcriptional networks	Circadian rhythms, life-cycle control	Chlorophyta, stramenopiles
BLUF-domain proteins	Blue	cNMP signaling, redox-sensitive regulators	Photobehavior, metabolic reprogramming	Stramenopiles, Chlorophyta
Type-1 rhodopsins	Blue–green	Ion fluxes, cNMP pathways	Phototaxis, photophobic responses	Dinoflagellates, cryptophytes, Chlorophyta
Phytochromes	Red/far-red	MAPK cascades, transcriptional reprogramming	Spectral acclimation, growth control	Chlorophyta, stramenopiles
Neochromes	Blue + red	Integrated kinase signaling modules	Phototropism, signal integration	Selected chlorophytes
UVR8	UV-B	ROS signaling, transcriptional regulation	UV acclimation, photoprotection	Selected chlorophytes

Note: Representative lineages are shown for illustrative purposes and do not imply exhaustive phylogenetic coverage.

Many algal photoreceptors show combinatorial domain architecture. For example, stramenopile aureochromes fuse a light-oxygen-voltage domain (LOV) sensor to a basic leucine zipper (bZIP) transcription factor, coupling light perception directly to transcriptional control within a single protein (Takahashi et al. 2007; Schellenberger Costa et al. 2013; Kroth et al. 2017; Coesel 2024). In other systems, including many green algae, light perception and downstream transcriptional responses are mediated by separate proteins linked through signaling cascades, enabling flexible rewiring of outputs (Mittag et al. 2005; Petersen et al. 2021).

### Integration of receptor input with photosynthetic excitation and redox signals

**Figure 4:**
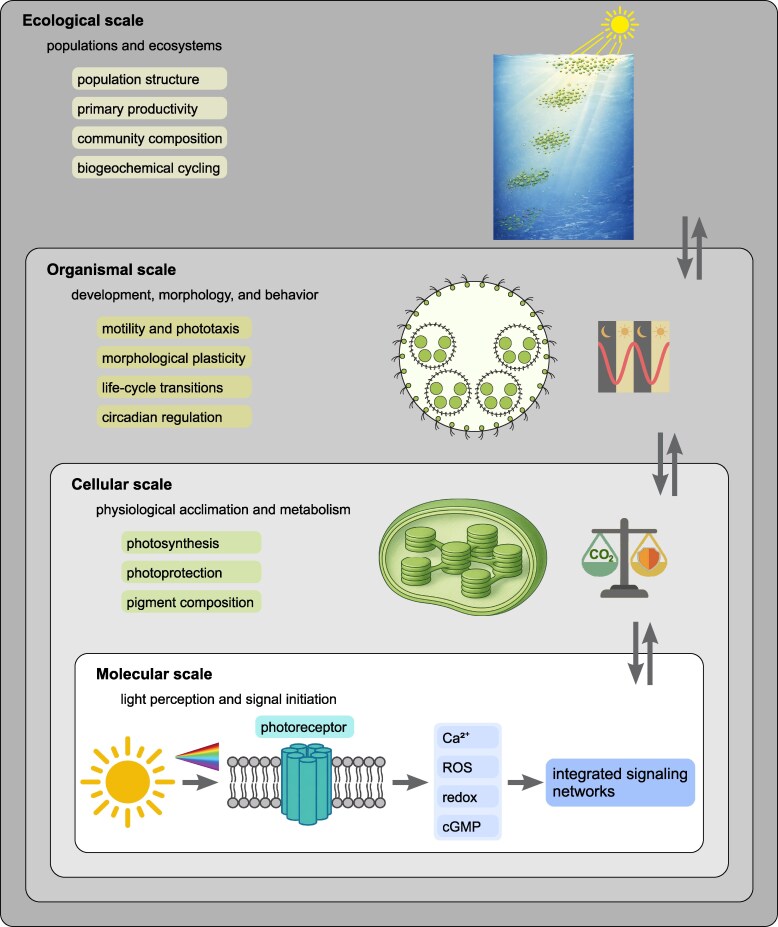
Scaling of light responses from molecules to ecosystems. Conceptual overview illustrating how light-dependent processes in algae propagate across biological scales, linking molecular light perception to ecosystem-level consequences. At the molecular scale, photons of different spectral qualities are perceived by diverse photoreceptors, initiating intracellular signaling cascades mediated by conserved second messengers such as Ca^2+^, ROS, redox signals, and cNMPs (eg cGMP), which converge into integrated intracellular signaling networks. These signals are processed at the cellular scale to regulate photosynthetic acclimation, photoprotection, pigment composition, and metabolism. Cellular light acclimation involves a dynamic trade-off between carbon gain and stress protection, illustrated by the balance symbol. At the organismal scale, integrated light signaling governs morphology, motility and phototaxis, circadian regulation, and life-cycle transitions, thereby shaping growth strategies and behavioral responses. These organism-level traits scale upward to ecological outcomes, influencing population structure and vertical distributions along light gradients, community composition, primary productivity, and biogeochemical cycling within aquatic and terrestrial ecosystems. Bidirectional arrows between levels indicate reciprocal feedbacks linking environmental light regimes, cellular signaling states, and organismal responses. The conceptual framework summarized in this figure was synthesized from information presented in the literature cited throughout this review.

In algae, photoreceptors operate alongside photosynthetic photochemical sensing. Photosynthetic electron transport, lumen pH, reactive oxygen species (ROS) formation, and the redox state of key carriers act as intrinsic metabolic sensors, generating regulatory signals even without dedicated photoreceptor proteins (Rockwell et al. 2014; Foyer 2018; Riaz et al. 2022; Hallmann 2025). Multiple spectral entry points (eg rhodopsins, cryptochromes, phototropins, phytochromes, and UVR8) converge on common intracellular signaling nodes, including Ca^2+^ dynamics, cyclic nucleotides (cNMPs), ROS/redox nodes, and chloroplast-to-nucleus retrograde signaling (Fig. 3) (Hegemann 2008; Rockwell and Lagarias 2020; Hallmann 2025). Thus, spectral, directional, and temporal inputs become coupled to excitation pressure and redox balance, linking light perception to cellular responses (Fig. 4) and enabling context-dependent decisions rather than fixed stimulus–response rules.

### Lineage-specific architectures as variations of a common sensing logic

**Figure 5:**
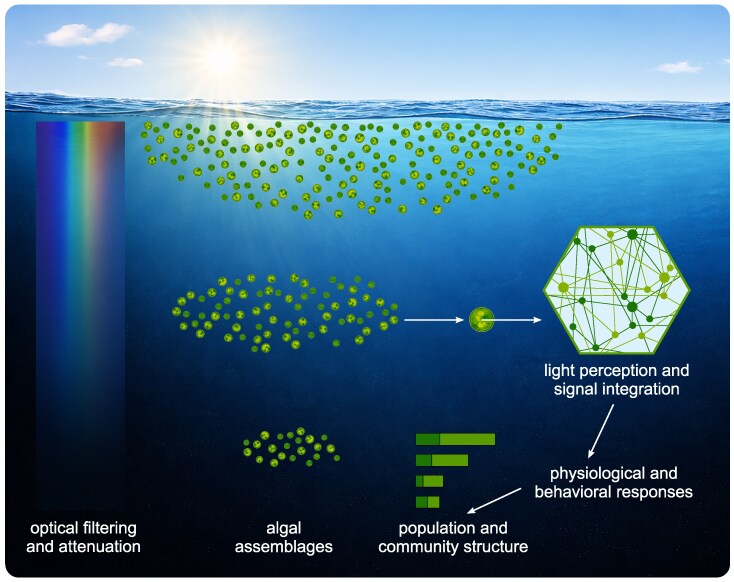
From light environment to ecological patterns. Solar radiation entering the water column is spectrally filtered and attenuated by the optical properties of the atmosphere and water, including wavelength-dependent scattering, absorption by pure water, CDOM, and algal pigments, thereby generating structured underwater light fields. Algal lineages within assemblages distributed along these gradients perceive the modified spectral and intensity information through diverse photoreceptors and integrate these signals at the cellular level. The resulting signaling states give rise to physiological and behavioral responses, including changes in photosynthetic performance, phototactic movement, stress acclimation, and cell-cycle regulation. At larger spatial and temporal scales, these responses propagate to population and community levels, shaping population distributions and community structure and generating characteristic ecological patterns along aquatic light gradients while linking optical forcing to biogeochemical processes. Thus, optical filtering of the light environment ultimately influences the organization and functioning of aquatic ecosystems. The conceptual framework summarized in this figure was synthesized from information presented in the literature cited throughout this review.

Environmental optics imposes strong, predictable filters on light fields. Atmospheric filtering, water, dissolved substances, particles, depth-dependent attenuation, turbidity, and colored dissolved organic matter (CDOM) together generate structured gradients in light intensity and spectral composition (Figs. 1 and 5) (Pope and Fry 1997; Morel and Maritorena 2001; Kirk 2010; Nima et al. 2019; Oelker et al. 2022; Hallmann 2025). Algal assemblages along these gradients contain lineages that perceive modified spectral and intensity signals through diverse photoreceptors and integrate them at the cellular level. The resulting signaling states translate into physiological and behavioral responses, including photosynthetic adjustment, phototactic movement, stress acclimation, and cell-cycle regulation. Across scales, these outputs can influence population distributions and community structure, although direct causal links from intracellular signaling to community structure remain limited. Photoreceptor complements and downstream wiring differ among lineages, but recurring functional motifs are evident (Table S1). Rhodopsin-based systems frequently dominate rapid behavioral loops, including phototactic and photophobic/photoavoidance responses, in motile green algae (Govorunova et al. 2004; Sineshchekov et al. 2009). Across diverse algal lineages, blue/UV-A flavin-based receptors commonly couple to transcriptional and physiological acclimation, including circadian entrainment and stress programs (Mittag et al. 2005; Beel et al. 2012; Petersen et al. 2021). Bilin-based phytochrome families exhibit particularly broad spectral tuning in several algal lineages compared with land plants, consistent with exploiting spectral gradients created by depth and shading (Rockwell et al. 2006, 2014; Duanmu et al. 2014; Rockwell and Lagarias 2020). UVR8 provides a conserved UV-B-sensing module, whose photochemical mechanism is best characterized in land plants, whereas algal UVR8 signaling and downstream circuitry appear more lineage-variable and remain less extensively resolved (Rizzini et al. 2011; Jenkins 2014; Allorent et al. 2016; Depaepe et al. 2023). These architectures shape niche performance rather than simple habitat labels. Depth-dependent filtering structures vertical communities and the deep chlorophyll maximum, where multiple lineages converge under low light and narrow spectral windows while coupling light responses to nutrient gradients and mixing (Morel and Maritorena 2001; Falkowski and Raven 2007; Kirk 2010). In extreme habitats (snow/ice, deserts, geothermal and hypersaline systems), the same principles apply: Strong UV/blue exposure and rapid fluctuation select for tight coupling of sensing to photoprotection, antioxidant defenses, and pigment-based screening (Sommaruga 2001; Leya 2013; Karsten and Holzinger 2014; Haniewicz et al. 2018; Chantzistrountsiou et al. 2023).

Taken together, the preceding examples support 3 recurring themes that are used here to organize the discussion of algal light sensing: (i) spectral specialization via modular receptor architectures, (ii) continuous integration of receptor signals with photosynthetic excitation/redox state, and (iii) lineage-specific wiring as variations on a common regulatory framework that maps optical constraints onto physiology, behavior, and life-cycle control. These themes synthesize established findings from comparative photoreceptor biology, photosynthetic redox regulation, and algal photoecology (Hegemann 2008; Kirk 2010; Petroutsos et al. 2016; Duanmu et al. 2017; Rockwell and Lagarias 2020; Petersen et al. 2021; Kreimer et al. 2023; Vierock and Hegemann 2023; Woodhams et al. 2023), while serving as the conceptual transition to the following sections. Table 1 summarizes receptor distributions; the main text emphasizes integration and functional outcomes.

## Multiscale integration through shared intracellular signaling networks

Intracellular signaling links heterogeneous light inputs to coordinated responses. In several well-studied algal systems, photoreceptor and redox inputs converge on recurrent messenger classes, including Ca^2+^, ROS/redox signals, cNMPs (cAMP/cGMP), and mitogen-activated protein kinase (MAPK) cascades (Dietz et al. 2016; Petroutsos et al. 2016; Mittler 2017; Wheeler 2017). The level of evidence, however, differs among modules and lineages. Comparative genomic analyses support Ca^2+^-signaling components across green, red, and secondary-plastid-containing algae, whereas direct functional evidence for light-dependent Ca^2+^ signaling is strongest in chlorophytes, especially *Chlamydomonas* and *Haematococcus* (Litvin et al. 1978; Harz and Hegemann 1991; Wheeler 2017; Kreimer et al. 2023). Cyclic nucleotide signaling is functionally documented in selected green algae and picoeukaryotes and is further supported by enzyme rhodopsins in several algal systems (Moulager et al. 2007, 2010; Tian et al. 2018; Mukherjee et al. 2019; Tsunoda et al. 2021; Vierock and Hegemann 2023). ROS/redox, MAPK, and retrograde modules are widely represented in algal genomes and stress-response datasets, but direct coupling to defined photoreceptors has been resolved mainly in model systems (Fischer et al. 2007; Miller et al. 2010; Dietz et al. 2016; Petroutsos et al. 2016; Kalapos et al. 2019; Gomez-Osuna et al. 2020). Accordingly, evolutionary origin and lifestyle should be treated as interacting axes: Phylogeny shapes available toolkits, whereas lifestyle and cellular organization shape their deployment for behavior, acclimation, photoprotection, or development.

In several algal systems, photoreceptor abundance, phosphorylation state, stability, and degradation are themselves modulated by intracellular signaling and acclimation state, illustrating reciprocal feedback regulation between photoreceptors and downstream signaling networks (Fig. 3) (Trippens et al. 2012; König et al. 2017; Böhm et al. 2019; Jaubert et al. 2022; Vierock and Hegemann 2023; Wolfram et al. 2023, 2024; Im et al. 2024b). In photosynthetic algal cells, the chloroplast is a major source of light-dependent metabolic signals and a major site of signal integration, linking photosynthetic excitation status to retrograde control of nuclear gene expression.

### Convergence of receptor and redox signals

Activation of distinct receptor classes (eg rhodopsins, phototropins, cryptochromes, phytochromes, and UVR8) can converge on shared second-messenger nodes. Photosynthetic electron transport itself can act as a conditional sensory module, because excitation pressure, plastid redox poise, and lumenal pH continuously generate regulatory information alongside photoreceptor-derived cues (Dietz et al. 2016; Petroutsos et al. 2016; Mittler 2017; Vierock and Hegemann 2023). These inputs are tightly coupled, placing spectral information in the context of metabolic state, consistent with the dual-input architecture outlined above. This integrated logic is particularly evident when nutrient limitation or low temperature constrains carbon assimilation: Electron transport becomes over-reduced and metabolic signals can mimic or amplify high-light signaling, triggering similar acclimation responses (Queval and Foyer 2012; He et al. 2015; Roncel et al. 2016; Borbély et al. 2022; Ye et al. 2022). A central component of this redox-linked signaling is ROS.

### ROS as a regulated signaling intermediate, not just a stress marker

Light-dependent ROS production is often framed as damage, but in algae it is better understood as a tunable metabolic signal superimposed on toxicity risk. The boundary between signaling and damage, however, remains poorly defined. The chloroplast is the primary site of ROS generation under fluctuating or excess light, producing singlet oxygen (^1^O_2_), superoxide (O_2_^−^•), and hydrogen peroxide (H_2_O_2_) when electron flow and downstream metabolic sinks are imbalanced (Dietz et al. 2016). Excess ROS can cause photoinhibition, but constrained ROS pulses also operate as retrograde signaling cues, activating nuclear programs that rebalance photosystem function, antioxidant capacity, and pigments (Apel and Hirt 2004; Fischer et al. 2007; Mittler 2017).

Algal studies support ROS-dependent acclimation, although the relevant ROS species and signaling routes differ among lineages. In *Chlamydomonas*, chloroplast-derived singlet oxygen has been implicated in chloroplast-to-nucleus retrograde signaling linked to acclimation (Fischer et al. 2007), whereas in diatoms high light induces oxygen photoreduction, ROS production, and ROS-scavenging responses (Waring et al. 2010). These pathways overlap with photoreceptor signaling, linking spectral perception to photosynthetic redox state and stabilizing responses under fluctuating light (Petroutsos et al. 2016; Mittler 2017; Vierock and Hegemann 2023).

A characteristic network feature is reciprocal coupling: Ca^2+^ regulates ROS-generating enzymes, while ROS modulates Ca^2+^ channel activity, forming feedback loops implicated in light-stress acclimation (Stael et al. 2012; Exposito-Rodriguez et al. 2017). These feedbacks support graded responses, from reversible acclimation to protective shutdown, depending on the duration and amplitude of excitation imbalance, and place Ca^2+^ and cNMPs at the interface of rapid signaling and longer-term physiological adjustment.

### Ca^2+^ and cNMPs as integrators

Ca^2+^ links light perception to rapid behavior and longer-term physiology. Light-induced photoreceptor potentials linked to algal phototaxis, including a regenerative response dependent on extracellular Ca^2+^, were first demonstrated in *Haematococcus pluvialis* (Litvin et al. 1978). In *C. reinhardtii*, Ca^2+^ signals are spatially organized near the eyespot and peri-flagellar region; voltage-gated Ca^2+^ channels translate channelrhodopsin-driven depolarization into rapid Ca^2+^ influxes (Harz and Hegemann 1991; Wheeler 2017; Kreimer et al. 2023). These transients determine flagellar beating asymmetry and thus phototactic steering (Kamiya and Witman 1984; Wakabayashi et al. 2009; Kreimer et al. 2023).

By analogy with broader plant Ca^2+^ signaling and supported by emerging algal data, chloroplast and cytosolic Ca^2+^ dynamics regulate photosynthetic enzyme activity, starch metabolism, and gene expression in response to light fluctuations and osmotic perturbations (Dodd et al. 2010). Many algal lineages encode a plant-like yet lineage-specific Ca^2+^ signaling toolkit (EF-hand sensors, CDPKs, CaMK-like components) with diversification in domain architectures and family expansions (Wheeler 2017). Ca^2+^ signals encode information through their spatial localization, the amplitude of concentration transients, and the frequency of Ca^2+^ spikes, allowing the same ion to regulate distinct outputs through different decoding mechanisms.

cNMPs provide a second rapid integrator for reversible physiological control. In several algal systems, cAMP/cGMP signaling can be light-modulated indirectly, but also directly via enzyme rhodopsins: Rhodopsin-guanylyl cyclases and rhodopsin-phosphodiesterases convert photons into immediate changes in cyclic nucleotide pools (Moulager et al. 2007, 2010; Tian et al. 2018; Mukherjee et al. 2019; Tsunoda et al. 2021; Vierock and Hegemann 2023). These receptors couple sensory input directly to second-messenger output, enabling real-time regulation of cNMP-controlled processes, including photomovement, transcriptional programs, and cell-cycle transitions (Moulager et al. 2007, 2010; Saegusa and Yoshimura 2015; Jansen et al. 2017; Mukherjee et al. 2019; Tsunoda et al. 2021).

In *Ostreococcus*, cAMP oscillates with the light–dark cycle and regulates cyclin A expression via interaction with the retinoblastoma (RB) protein, directly connecting photoperiod to cell division control (Moulager et al. 2007, 2010). Light-dependent cGMP dynamics have likewise been implicated in photomovement and osmotic signaling (Holland et al. 1997; Kreimer 2009; Saegusa and Yoshimura 2015; Jansen et al. 2017; Kreimer et al. 2023). Ca^2+^ and cNMPs thus serve as central intermediates through which diverse sensory inputs converge on common downstream pathways.

### Network properties: feedback, amplification, modularity, and gain control

MAPK cascades mediate signal amplification and integration during environmental transitions. In microalgae, MAPKs respond to salinity, osmotic shifts, oxidative stress, and light transitions, forming stress-activated modules that resemble ERK/MEK and p38/HOG-like systems but include algal-specific paralogs and rewired regulatory connections (Chen and Thorner 2007; Colcombet and Hirt 2008; Miller et al. 2010; Rodriguez et al. 2010; Hamel et al. 2012; Kalapos et al. 2019; Gomez-Osuna et al. 2020). In algae and by comparison with broader plant stress-signaling systems, ROS and Ca^2+^ can converge on MAPKs, enabling crosstalk between excitation/redox signals and transcriptional reprogramming (Apel and Hirt 2004; Mittler et al. 2004; Mühlenbock et al. 2008; Fischer et al. 2013; Jalmi and Sinha 2015).

Three properties are especially relevant:

- **Feedback and amplification control**: Reciprocal ROS–Ca^2+^ coupling and redox-dependent modulation of signaling nodes regulate signal amplification and stabilize photosynthetic function while permitting rapid switching when thresholds are crossed (Stael et al. 2012; Exposito-Rodriguez et al. 2017).
- **Modularity**: Receptor-specific entry points feed into partially separable downstream modules (motility, photoprotection, cell cycle), reducing interference while enabling recombination of outputs across lineages.
- **Context-dependent gain control**: Nutrient status, temperature, and salinity tune network gain, shifting the balance between carbon gain and protection.

### Environmental modulation of light signaling

Light responses depend on environmental context: Nutrient limitation, temperature, and salinity modulate both metabolic signal generation and the interpretation of photoreceptor cues. Most mechanistic studies still manipulate single factors, whereas natural habitats impose correlated changes, leaving interaction effects poorly resolved. Recent studies increasingly demonstrate that nutrient availability, temperature, salinity, and light interact through shared photoreceptor-, redox-, and stress-signaling networks, producing responses that cannot be predicted from individual factors alone (Shetty et al. 2019; Gao et al. 2024a; Hemker et al. 2024). The result is photophysiological plasticity across ecosystems, from oligotrophic gyres to iron-limited polar waters and hypersaline ponds (Behrenfeld et al. 2006; Raven 2011).

#### Nutrient availability and carbon status

Fe limitation directly constrains electron transport capacity (PSI, cytochrome b_6_f, ferredoxin), forcing antenna remodeling and substitution of Fe-dependent enzymes, thereby altering excitation pressure and redox signaling under a given light regime (Sunda and Huntsman 1997; Strzepek and Harrison 2004; Allen et al. 2008; Morrissey and Bowler 2012; Sutak et al. 2020). Recent evidence further suggests that nutrient availability can intersect directly with photoreceptor-mediated signaling pathways; in marine diatoms, the cryptochrome PtCPF1 contributes to high-temperature acclimation by coordinating iron and phosphorus uptake, illustrating direct coupling between photoreceptor signaling, nutrient homeostasis, and high-temperature acclimation (Gao et al. 2024a). Carbon limitation in *Chlamydomonas* activates the carbon-concentrating mechanism (CCM) and co-induces LHCSR genes through CIA5/CCM1, with phototropin modulation linking carbon status to photoprotection (Fang et al. 2012; Petroutsos et al. 2016; Vierock and Hegemann 2023).

#### Temperature

Cold slows enzymatic sinks for electrons, increasing photoinhibition risk even at moderate light (“photostasis”), selecting for enhanced antioxidants and cyclic electron flow; heat stress can likewise exacerbate photooxidative pressure, requiring temperature-dependent tuning of photoprotection and repair pathways (Raven and Geider 1988; Huner et al. 1993; Mock and Hoch 2005; Morgan-Kiss et al. 2006; Szyszka-Mroz et al. 2015).

#### Salinity and osmotic stress

In *Dunaliella salina*, osmotic acclimation (glycerol accumulation) and carotenoid synthesis (β-carotene) are shaped by both osmotic and light inputs, with MAPK-like and ROS-dependent signaling integrating ionic stress into redox and photoreceptor pathways (Ben-Amotz et al. 1982; Chitlaru and Pick 1991; Liska et al. 2004; Ben-Amotz et al. 2009; Shabala and Mackay 2011). More broadly, salinity and osmotic stress responses involve extensive crosstalk between ROS signaling, antioxidant defenses, and light-responsive regulatory pathways, highlighting their integration with photophysiological acclimation processes (Shetty et al. 2019). Similarly, combined exposure to elevated salinity and high irradiance can elicit responses distinct from those observed under individual stress factors, emphasizing the importance of multifactorial environmental interactions in algal acclimation (Hemker et al. 2024).

#### UV and high irradiance

UV-B signaling is best characterized in land plants, where UVR8 acts through constitutively photomorphogenic 1 (COP1)-related signaling components, but algal evidence also supports UVR8-dependent photoprotection in *Chlamydomonas*. More broadly, UV-B exposure can activate DNA repair by photolyases, mycosporine-like amino acid (MAA) biosynthesis, and antioxidant defenses; CDOM variability modulates UV exposure and thus shapes local selection on these protective networks (Sommaruga 2001; Rizzini et al. 2011; Rastogi and Incharoensakdi 2013; Tilbrook et al. 2013; Allorent et al. 2016; Tilbrook et al. 2016).

### Signal-linked chloroplast adaptations

At the chloroplast level, adaptive changes reflect outputs of photoreceptor–redox signaling. Photoreceptors regulate pigment composition and photosystem balance (Rochaix 2011), while excitation/redox-derived signals tune metabolic allocation including diversion of reducing power into storage lipids under high light or nutrient stress (Hu et al. 2008; Msanne et al. 2012). Structural and positional changes, including chloroplast movement and thylakoid remodeling, provide additional downstream outputs that adjust excitation pressure and photosynthetic capacity over short and longer timescales. Structural plasticity also contributes to light acclimation, but the evidence base differs among processes. Thylakoid and photosystem remodeling are well-documented algal acclimation responses that adjust light-harvesting capacity to metabolic demand (Minagawa 2011; Rochaix 2011; Goss and Lepetit 2015; Minagawa and Tokutsu 2015; Ostermeier et al. 2024). By contrast, photoreceptor-mediated chloroplast movement, including accumulation under low light and avoidance under high light, is best established in land plants (Wada et al. 2003; Wada 2013); in algae, comparable geometry-based control is likely restricted to taxa with suitable chloroplast morphology, such as forms with large or flattened plastids.

Taken together, available evidence from well-studied algal systems indicates that light responses frequently converge on a shared set of intracellular messengers, including ROS/redox signals, Ca^2+^, cNMPs, and MAPK cascades. However, the molecular toolkits, coupling strengths, and experimentally resolved pathways differ substantially among lineages, and functional evidence remains strongest for chlorophytes and selected stramenopiles. These differences should therefore be interpreted along 2 interacting axes: Evolutionary history shapes the available signaling components, whereas lifestyle and cellular organization shape their deployment for rapid behavior, metabolic acclimation, development, or stress protection. Environmental factors (nutrients, temperature, salinity, UV) tune network gain and configuration, matching light-dependent outputs to metabolic capacity and stress tolerance in fluctuating habitats (Behrenfeld et al. 2006; Falkowski and Raven 2007; Dietz et al. 2016; Petroutsos et al. 2016; Mittler 2017).

## Functional outputs of integrated light signaling: photosynthesis and photoprotection

Photosynthetic algae collectively contribute roughly half of global carbon fixation and thus couple light-driven physiology directly to biogeochemical fluxes (Field et al. 1998; Falkowski and Raven 2007). At the cellular level, the central constraint is regulatory: Absorbed photons must match electron transport capacity and downstream metabolic demand. Integrated light signaling coordinates photosynthetic capacity, antenna size, and photoprotection through interactions between photoreceptor inputs and chloroplast-derived redox signals.

### Regulatory control of photosynthetic capacity and chloroplast plasticity

Photosynthesis begins with photon capture by PSII/PSI-associated antenna complexes and other lineage-specific antenna systems, including membrane-embedded light-harvesting complexes, phycobilisomes associated with the stromal surface of thylakoid membranes in red algae and Glaucophyta, and luminal phycobiliproteins in Cryptophyta (Frank and Brudvig 2004; Nelson and Ben-Shem 2004; Ozawa et al. 2009; Frank and Cogdell 2012; Nickelsen and Rengstl 2013; Willows 2020). The key regulatory variable is the balance between light harvesting, electron transport, and metabolic sinks. Excitation energy drives charge separation at PSII (reaction center P680) and PSI (reaction center P700), generating NADPH and ATP that fuel carbon fixation and broader metabolism (Frank and Brudvig 2004; Frank and Cogdell 2012).

Because ATP and NADPH supply multiple pathways, light responses necessarily reallocate cellular resources and reshape cellular C:N balance (Huppe and Turpin 1994). Photoreceptors and chloroplast retrograde signals jointly regulate photosynthetic gene expression, antenna composition, and metabolic enzyme capacity, with circadian systems coordinating translation and biosynthesis across diel cycles (Kirk and Kirk 1985; Mittag et al. 2005; Rochaix 2011).

A major long-term output is chloroplast and thylakoid remodeling. In algae, thylakoid and photosystem remodeling contribute to photoacclimation by adjusting antenna organization, photosystem balance, and light-harvesting capacity to metabolic demand (Minagawa 2011; Rochaix 2011; Goss and Lepetit 2015; Minagawa and Tokutsu 2015; Ostermeier et al. 2024). Photoreceptor-mediated chloroplast movement provides rapid control of light absorption in land plants (Wada et al. 2003; Wada 2013), whereas comparable positioning-based control has been documented or inferred only for a limited subset of algae and is likely most relevant in taxa with suitable chloroplast morphology, such as forms with large or flattened plastids. Together, these structural and positional responses tune photon capture to metabolic demand.

Photosynthesis is also tightly linked to storage allocation. Under high light or nutrient stress, excess reducing power and carbon are diverted into triacylglycerol accumulation, whereas low light favors investment into pigments and membranes (Hu et al. 2008; Msanne et al. 2012). When metabolic sinks cannot balance excitation, excess energy is dissipated through photoprotective mechanisms.

### Photoprotection as feedback control

Photoprotection comprises strategies that dissipate excess excitation, regulate light absorption, and repair photodamage under fluctuating or high irradiance (Fig. 2), with core concepts established across oxygenic photosynthetic organisms and lineage-specific implementations in algae (Demmig-Adams and Adams 1996; Niyogi 1999; Allorent and Petroutsos 2017; Vecchi et al. 2020). When absorbed energy exceeds utilization capacity, ROS levels rise, imposing both risk and information (Asada 2006). Photoprotection relies on feedbacks in which lumen pH, redox state, and ROS help trigger compensatory responses.

NPQ is a central terminal effector that converts excitation surplus into heat, protecting PSII (Ruban 2016; Vecchi et al. 2020). NPQ is strongly gated by thylakoid lumen acidification (ΔpH) and modulated by the xanthophyll cycle (violaxanthin→zeaxanthin), linking energy dissipation directly to the proton motive force and excitation pressure (Peers et al. 2009; Dinc et al. 2016). A key feature is that different lineages use the same underlying signal, the thylakoid proton gradient (ΔpH), in different ways: Chlorophytes primarily use LHCSR proteins, diatoms employ the diatom stress-related LHC (LHCX) protein, and red algae show mechanistically distinct (often phycobilisome-associated) NPQ variants (Peers et al. 2009; Bailleul et al. 2010; Taddei et al. 2016, 2018).

In addition to NPQ, several algal lineages tune excitation distribution via state transitions, reallocating energy between PSII and PSI to stabilize electron flow under changing spectra and intensities (Minagawa 2011; Minagawa and Tokutsu 2015). These rapid responses are complemented by slower photoacclimation, including antenna remodeling, pigment shifts, and sustained accumulation of protective compounds that adjust system gain under persistent light regimes (Goss and Lepetit 2015).

Damage control is integrated into this regulatory logic. PSII photodamage—especially to the PSII reaction center protein D1—is countered by the PSII repair cycle, and photoinhibition can be viewed as a regulated relief valve for excitation pressure constrained by repair capacity (Aro et al. 1993; Raven 2011; Tikkanen and Aro 2014). Alternative electron transport routes further buffer the redox state: Cyclic electron flow adjusts the ATP/NADPH balance, while the Mehler reaction and plastid terminal oxidase (PTOX) can divert electrons to prevent overreduction (Cardol et al. 2011; Johnson 2011; Alric and Johnson 2017; Peltier et al. 2024). ROS detoxification (superoxide dismutase, peroxidases, catalase, glutathione-dependent systems) completes the control loop by limiting ROS accumulation while allowing ROS-dependent signaling to proceed (Asada 2006; Foyer et al. 2017).

Behavioral photoprotection adds a whole-cell actuator in motile algae: Photoreceptors can trigger negative phototaxis or photoshock responses that physically move cells away from damaging irradiance (Hegemann 2008; Allorent and Petroutsos 2017).

### Pigment biosynthesis and antenna tuning

Pigment systems function as both light-harvesting components and regulators of light use (Fig. 2) (Begum et al. 2016; Geuer et al. 2023; Ángeles et al. 2025). Light regulates chlorophyll and carotenoid biosynthesis to adjust antenna size, spectral tuning, and photoprotection (Croce and van Amerongen 2014; Begum et al. 2016; Ángeles et al. 2025). Chlorophyll variants differ in their physicochemical and spectral properties (Kobayashi et al. 2013). Spectral complementarity across chlorophylls, carotenoids, and (in red-lineage systems) phycobiliproteins supports efficient light use, but regulation primarily involves changes in antenna size and protective pigment composition.

Chlorophyll content typically increases under low to moderate light and declines under very high irradiance (“chlorophyll downregulation”), reducing over-absorption through coordinated regulation of biosynthesis and turnover (Falkowski 1980; Rochaix 2011). Many green algae adjust chlorophyll a:b ratios to tune antenna properties (Anderson and Chow 2002). Unlike higher plants, many algae retain both light-dependent protochlorophyllide oxidoreductase (POR) and light-independent dark-operative POR, allowing chlorophyll synthesis in darkness while maintaining light-dependent regulation of POR expression and activity (Neuberger 1980; Tanaka and Tanaka 2007; da Silva and Lombardi 2020; Willows 2020).

In addition to chlorophyll-based antenna tuning, carotenoids contribute to harvesting (eg fucoxanthin and peridinin) and protection. Blue-light signaling promotes carotenoid-pathway gene expression (eg phytoene synthase and phytoene desaturase), supporting accumulation of protective carotenoids, including secondary pigments such as astaxanthin under high light or nutrient stress (Han et al. 2013; Lee et al. 2018; Zarekarizi et al. 2023; Debnath et al. 2024). UV exposure further induces carotenoids and UV-screening compounds such as MAAs; cyanobacteria and some algae produce scytonemin as an additional sunscreen pigment (Balskus and Walsh 2010; Carreto and Carignan 2011; Mutschlechner et al. 2022). These responses operate on longer timescales and reduce excitation pressure and oxidative risk while preserving productivity.

Integrated light signaling regulates photosynthesis through interacting fast and slow responses: Rapid responses (NPQ, state transitions, chloroplast movement, alternative electron flow) stabilize excitation and redox state, whereas slower changes (antenna and pigment reconfiguration, thylakoid restructuring, repair capacity, storage allocation) determine sustained performance under changing light conditions (Minagawa 2011; Rochaix 2011; Goss and Lepetit 2015; Ruban 2016).

## Light regulation of development and cell-cycle progression

In many algal systems, light regulates allocation among vegetative growth, division, reproductive differentiation, and survival programs. It integrates photosynthetic energy supply, photoreceptor-derived spectral and temporal information, circadian gating, and stress signals such as ROS. Division or reproduction proceeds only when light-driven metabolism and environmental conditions are favorable; otherwise cells delay division, arrest, or enter sexual programs that promote survival and genetic repair.

### Cell-cycle control: light gates commitment, division number, and timing

Control over the G1→S transition is especially strong in photoautotrophic algae, where light acts as both metabolic fuel and checkpoint input (Cross and Umen 2015; Zhao et al. 2022). During G1, cells grow and accumulate biosynthetic capacity; only after reaching a size/resource threshold can they enter S phase. In many green algae, including *C. reinhardtii*, this logic is implemented as multiple fission: An extended G1 growth phase is followed by rapid rounds of DNA replication and cytokinesis that produce 2^n^ daughter cells, with light intensity and photoperiod determining final mother-cell size and thus division number (Craigie and Cavalier-Smith 1982; Donnan and John 1983; Zachleder et al. 2002; Bišová and Zachleder 2014).

At the molecular level, cyclin–cyclin-dependent kinase (cyclin–CDK) modules and RB-related proteins implement this checkpoint. In *Chlamydomonas*, the late-G1 commitment point integrates size, nutrients, and light and becomes irreversible once crossed (Cross and Umen 2015; Cross 2020). Similar light and temporal coupling occurs in *Ostreococcus tauri*, where simplified cyclin–CDK networks gate S-phase entry (Corellou et al. 2005; Moulager et al. 2007, 2010). Macroalgal examples extend this logic beyond green algae: Diel cell-cycle progression occurs in *Gracilariopsis chorda*, and blue light regulates cell-cycle-related gene expression, growth, and gametogenesis in *Saccharina japonica* gametophytes (Lee et al. 2024; Xu et al. 2025b). In studied chlorophytes, blue light often delays G1→S, increasing commitment size and prolonging the cell cycle, whereas red light can shorten G1 and synchronize division to diel rhythms (Oldenhof et al. 2004a, 2004b, 2006). These effects involve blue-light receptors, including cryptochromes and phototropins, and possibly phytochrome-like systems, although molecular identities remain incompletely resolved in several algal systems (Beel et al. 2012; Fortunato et al. 2016; Rockwell and Lagarias 2017). Beyond chlorophytes, additional receptor solutions, such as aureochromes in stramenopiles, indicate repeated evolutionary coupling of light perception to division control (Kottke et al. 2018; Hallmann 2025).

Stress-derived signals can override light-driven growth cues. High light or UV elevates ROS, which can slow or arrest the cell cycle; in *Chlamydomonas*, ROS also intersect with sexual differentiation pathways (Mullineaux et al. 2018; Pokora et al. 2022). Light can therefore both promote division (via energy supply and timing cues) and inhibit it (via oxidative signaling), depending on the balance between metabolic capacity and excitation stress.

### Sexual reproduction and life-cycle transitions: light sets competence, synchrony, and repair logic

Light regulates the sexual cycle at 3 recurring control points: gamete formation, mating competence, and post-zygotic development (germination and early development) (Pan et al. 1997; Huang and Beck 2003; Goodenough et al. 2007; Nishihama and Kohchi 2013; Bachy et al. 2022; Jiang et al. 2025). In green algae, blue-light pathways are frequently linked to gametogenesis and zygote programs. In *Chlamydomonas*, phototropin contributes to gametogenesis and germination, and an animal-like cryptochrome (aCRY) participates in germination and early zygotic development (Huang and Beck 2003; Zou et al. 2017).

Nitrogen deficiency is a dominant trigger of sexual reproduction in *Chlamydomonas*, whereas in multicellular volvocine algae such as *Volvox*, this process is primarily regulated by extracellular sex-inducing signals. Across these systems, light often synergizes with nutrient status and metabolic/redox stress to induce gametes (Huang and Beck 2003; Goodenough et al. 2007; Harris et al. 2009; Mikami 2022). Under high irradiance, ROS can promote sexual reproduction, whereas low light prolongs vegetative growth and delays sexual transitions (Permann et al. 2022). UV-B provides an additional damage-associated cue: DNA damage may bias cells toward sex, increasing genetic variability and enabling recombination-based repair (Häder and Sinha 2005; Roleda et al. 2006).

Photoperiod provides population-scale synchrony. In *C. reinhardtii*, short days (<12 h light) favor sexual reproduction, whereas long days favor vegetative proliferation (Mouget et al. 2009). In diatoms, light interacts with temperature and nutrient limitation (often including silicate) to trigger sexualization and auxosporulation, aligning reproduction with seasonal windows (Chepurnov et al. 2004, 2008; Mouget et al. 2009; Godhe and Rynearson 2017). In brown algae such as *Fucus*, photoperiod and spectral quality regulate gametogenesis and release, with spawning synchronized to tidal and light cycles and often occurring in the morning (Serrao et al. 1996; Pearson and Howard Brawley 1998; Coelho et al. 2007; Ruiz Martínez et al. 2024). Dinoflagellates can integrate diel and lunar light cues to coordinate sexual cycles and cyst formation (Figueroa et al. 2008; Bravo and Figueroa 2014).

At the gene-regulatory level, master regulators connect environmental inputs to cell-type programs: The *Chlamydomonas* minus dominance gene (MID) and volvocine GSP1 and GSM1 exemplify sex-related transcriptional control linked to light and nitrogen status (Ferris and Goodenough 1997; Umen and Olson 2012; Umen 2018). In *V. carteri*, environmental light modulates the production and perception of an extracellular sex-inducing signal (“sex-inducer”), coupling photic context to intercellular signaling (Kirk 1998; Hallmann 2011). Across systems, cells invest in vegetative growth under favorable conditions or switch to recombination and survival when light-driven stress and resource limitation increase.

### Development and photomorphogenesis: light as an instructive cue

Across micro- and macroalgae, light controls differentiation, morphogenesis, and life-cycle transitions, linking them to ecological adaptation (Dring and Lüning 1983; Dring 1988). Photoperiodism is a dominant timing mechanism in many macroalgae, aligning these transitions with seasonal light regimes (Lüning 1990). In *Pyropia* spp. (formerly Porphyra), short days promote vegetative proliferation, whereas long days favor sexual reproduction and carposporophyte formation (Lüning 1990). In kelps (eg *Laminaria* and *Saccharina*), blue light and daylength regulate gametophyte development and fertility; in *Ulva*, irradiance and photoperiod jointly control spore release and thallus morphogenesis (Lüning 1990; Wichard et al. 2015).

Spectral quality can direct developmental responses. Blue light frequently drives morphogenetic programs; in diatoms such as *Phaeodactylum tricornutum*, blue light affects chloroplast ultrastructure and photosynthesis-related gene expression (Agarwal et al. 2023). In stramenopiles, aureochromes couple blue-light perception directly to transcription via their LOV–bZIP architecture (Takahashi et al. 2007; Banerjee et al. 2016; Heintz and Schlichting 2016; Mann et al. 2020; Coesel 2024; Im et al. 2024a). Red/far-red-responsive developmental responses in *Pyropia* suggest a role for bilin-dependent (phytochrome-like) signaling in algal development (Duanmu et al. 2014).

In volvocines, light can act as both a positional cue and a developmental signal. In *V. carteri*, blue-light-responsive programs differ between somatic and reproductive cell types; the vegetative-to-reproductive transition integrates photic input with oxidative and metabolic cues (Kirk and Kirk 1985; Hallmann 2011; Kianianmomeni and Hallmann 2014; Umen 2020). Related forms (eg *Gonium* and *Eudorina*) illustrate tight coupling of phototactic organization and early multicellular development (de Maleprade et al. 2020).

Developmental responses are often mediated by shared second messengers. Ca^2+^ and ROS have been implicated in gametogenesis, fertilization, and zygote development across taxa (Kim et al. 2025). In fucoid zygotes, tip-focused Ca^2+^ and ROS gradients coordinate polarization, with ROS coupled to localized Ca^2+^ influx (Love et al. 1997; Coelho et al. 2008a, 2008b). Comparable light-induced Ca^2+^ transients occur in *Chlamydomonas*, supporting the view that Ca^2+^ acts as a conserved photomorphogenetic and stress-signaling integrator (Pivato et al. 2023). In *Haematococcus*, blue light influences astaxanthin accumulation and carotenoid-pathway gene expression; cryptochrome-mediated blue-light signaling may contribute to this regulation (Lee et al. 2018; Zhang et al. 2022). MAPKs and cyclic nucleotide pathways provide additional points of integration linking light with nutrient and stress signals into morphogenetic outcomes (Fraikin et al. 2023).

### Circadian rhythms as temporal gates

Circadian clocks align metabolism, division, and reproduction to favorable times of day and anticipate daily light–temperature cycles (Mittag et al. 2005; Kuhlman et al. 2018). Across studied algal systems, circadian outputs include photosynthesis, nitrogen assimilation, starch metabolism, phototaxis, sporulation, and cell-cycle timing (Bruce 1972; Mittag 2001; Noordally and Millar 2015; Farré 2020). In *Chlamydomonas*, clocks modulate DNA replication and cell division (Mittag et al. 2005; Matsuo et al. 2008; Kamrani et al. 2018), whereas in *Ostreococcus*, circadian gating of S phase is coupled to the light–dark cycle and cAMP/RB-linked cell-cycle control (Moulager et al. 2007, 2010). Recent work in *P. tricornutum* further demonstrated circadian regulation of key physiological processes by the clock-associated protein RITMO1 (Manzotti et al. 2025). Inputs to the oscillator include photoreceptors, notably cryptochromes, and in some taxa LOV- or rhodopsin-type histidine kinases (Thommen et al. 2015). Dinoflagellates are notable for predominantly translational circadian control (McMurry and Hastings 1972; Hastings 2013; Jadhav et al. 2022), and nontranscriptional oscillators can contribute even in eukaryotic picoalgae (O'Neill et al. 2011). Together with photosynthetic output, photoreceptor input, and ROS/stress signals, clocks gate transitions such as S phase, gamete release, arrest, or sexual pathways into temporal windows that balance resource availability, stress avoidance, and survival.

## Behavioral strategies in algal light responses

Integrated light signaling translates optical cues into motility decisions that optimize photosynthetic gain and minimize photodamage (Fig. 2). Behavioral responses do not depend on isolated signaling pathways but draw on shared intracellular hubs (Fig. 3)—Ca^2+^, cNMPs, membrane voltage, and redox/ROS—allowing rapid switching between approach, avoidance, and reset states (Lenci and Colombetti 1978; Foster and Smyth 1980; Sasso et al. 2018; Leptos et al. 2023).

### Light-guided behavioral control: phototaxis, photokinesis, and avoidance responses

Motile microalgae deploy a small repertoire of light-dependent behaviors—photokinesis (speed modulation), phototaxis (directional movement), and photophobic/photoshock responses (rapid avoidance following abrupt irradiance changes; Fig. 2)—that together generate flexible responses rather than fixed stimulus–response patterns (Diehn et al. 1977; Häder 1979; Nultsch and Häder 1979, 1988; Feinleib 1980; Sgarbossa et al. 2002; Harris et al. 2009; Wakabayashi et al. 2021). These behaviors translate molecular photoreception and intracellular signaling into movement within environmental light gradients (Fig. 4) (Cortese and Wan 2021). Functionally, they determine which light regimes are favorable and how cells navigate toward them. Different algal lineages implement these functions through distinct sensory architectures. In *Chlamydomonas*, these behavioral modes are complemented by light-regulated surface adhesion and gliding, illustrating that photobehavior comprises several mechanistically distinct but physiologically integrated outputs (Kreimer et al. 2023).

Across lineages, photobehavior has evolved repeatedly, often with nonhomologous directional-sensing structures (eyespot-like organelles) in chlorophytes, euglenophytes, dinoflagellates, and cryptophytes (Hegemann and Deininger 2001; Dieckmann 2003; Kreimer 2009; Böhm and Kreimer 2021; Kreimer et al. 2023). This evolutionary diversity extends beyond eyespot architecture to the photoreceptors and transduction mechanisms themselves. Whereas channelrhodopsin-based phototactic signaling is directly established in chlorophytes and rhodopsin-based photobehavioral systems have been proposed or identified in other algal lineages, euglenophytes use flavin-based photoactivated adenylyl cyclases that regulate cyclic nucleotide signaling rather than relying on channelrhodopsin-mediated membrane depolarization (Iseki et al. 2002; Ntefidou et al. 2003). In chlamydomonad algae, directional input is generated by an eyespot apparatus in which carotenoid-rich chloroplast globule layers, specialized chloroplast-envelope regions, and a plasma-membrane patch containing channelrhodopsins act together as an optical antenna. This architecture produces a directional light signal through asymmetric illumination of the photoreceptor layer, with channelrhodopsins mediating the primary photoreceptor step in native *Chlamydomonas* phototaxis (Morel-Laurens and Feinleib 1983; Nagel et al. 2002; Sineshchekov et al. 2002; Nagel et al. 2003; Engel et al. 2015; Ueki et al. 2016; Böhm and Kreimer 2021; Ueki et al. 2022; Kreimer et al. 2023). In this system, the key architectural principle is prefiltering coupled to rapid photoreceptor-driven changes in membrane voltage, converting directional light information into a flagellar response.

Once directional information is detected, ion-based signaling translates it into changes in flagellar beating. In *Chlamydomonas*, asymmetric Ca^2+^ dynamics control the switch between symmetric and asymmetric ciliary beating, thereby implementing steering rather than simple propulsion mechanics (Kamiya and Witman 1984; Wakabayashi et al. 2009; Wan et al. 2014; Saegusa and Yoshimura 2015; Wan and Goldstein 2016; Woodhams et al. 2023). Mechanosensitive channels, cNMPs, and proton fluxes further tune excitability and response thresholds, particularly for photoshock-like avoidance behaviors (Yoshimura and Kamiya 2001; Fujiu et al. 2009; Kreimer 2009). Photobehavior proceeds through photoreceptor activation, rapid second-messenger or voltage changes, and selection of a motility mode (approach, avoid, reset). In addition to swimming behavior, light also controls surface-associated responses. In *C. reinhardtii*, blue and red light regulate flagellar adhesion to surfaces, enabling reversible light-dependent attachment and detachment of cells (Kreis et al. 2018; Catalan et al. 2023). Such light-regulated adhesion links photobehavior to surface colonization and microalgal biofilm formation, whose structure and physiology can be strongly shaped by light intensity (Gao et al. 2024b).

The same logic extends to larger organizational scales: Multicellular volvocine algae such as *Volvox* implement coordinated phototaxis by distributing steering across many somatic cells, demonstrating that behavior scales by reusing conserved signaling modules rather than introducing new control principles (Drescher et al. 2010; Woodhams et al. 2023). Recent work further suggests that light-dependent behavioral regulation can extend beyond individual or colonial coordination to population-level organization: In marine diatoms, phytochrome-mediated intercellular communication was shown to drive collective behavioral responses and reshape population structure under changing light conditions (Font-Muñoz et al. 2026). Beyond phytochrome- and channelrhodopsin-driven steering, other receptors (phototropins, cryptochromes, additional rhodopsins) modulate behavioral state via effects on eyespot organization, circadian timing, and stress responses (Huang and Beck 2003; Hegemann 2008; Beel et al. 2012; Petersen et al. 2021; Govorunova and Sineshchekov 2023). Genome editing and engineered rhodopsins now enable causal dissection of these mechanisms, while algal channelrhodopsins have become central optogenetic actuators for controlling cellular activity in heterologous systems (Shin et al. 2016; Govorunova et al. 2017; Greiner et al. 2017).

### Phototropism in macroalgae

Sessile macroalgae reorient growth toward favorable light, with blue light often serving as the dominant directional cue, consistent with a conserved reliance on short-wavelength signaling for phototropic growth (Rico and Guiry 1996; Takahashi and Mikami 2016; Takahashi and Mikami 2019). In brown algae, particularly fucoid zygotes, unilateral blue light can establish developmental polarity, inducing rhizoid formation on the shaded side and thereby defining the growth axis. In stramenopiles such as *Vaucheria*, aureochromes (LOV–bZIP) couple blue-light perception directly to transcriptional regulation and contribute to phototropic morphogenesis (Takahashi et al. 2007; Toyooka et al. 2011; Kroth et al. 2017). In several red macroalgae, phototropic phenomena occur despite the apparent absence of canonical phototropins or phytochromes, implying the existence of alternative photoreceptor solutions that remain mechanistically unresolved (Duanmu et al. 2014; Takahashi and Mikami 2016; Rockwell and Lagarias 2020).

## Ecological feedback and evolutionary variation of a common regulatory logic

Environmental light fields are mapped onto cellular states by an integrated sensing architecture that couples light harvesting and photoreceptor perception through shared signaling hubs (Figs. 3 and 5) (Kirk and Kirk 1985; Huang and Beck 2003; Grossman et al. 2004). Algal diversification can be interpreted as variation on a common regulatory framework: partly conserved and recurrent photoreceptive, chloroplast redox, and second-messenger modules driving physiological and behavioral outputs, with lineage- and habitat-specific rewiring of sensors, thresholds, and effector modules. The light environment itself is a major driver of this rewiring, reflected in the diversity of algal photic habitats.

### Habitat filters select network architecture, not just pigments

Algal lineages occupy almost every photic habitat—from freshwater and coastal waters to open oceans, polar snowfields, desert crusts, and geothermal springs (Raven and Giordano 2014). In the green lineage, phylogenomic analyses of streptophyte algae and embryophytes provide an evolutionary framework for comparing light-dependent photoprotection and stress-acclimation traits across aquatic, semiterrestrial, and terrestrial habitats (Wodniok et al. 2011). Across these environments, habitat acts as a selective filter on network tuning, shaping which wavelengths matter, how rapidly light fluctuates, how often cells experience overexcitation, and how tightly light responses must be coupled to nutrients and temperature.

A key physical constraint is spectral filtering with depth. Light declines exponentially, and red wavelengths attenuate rapidly, leaving predominantly blue–green light in deeper layers (Morel and Maritorena 2001; Falkowski and Raven 2007; Kirk 2010). This structure underpins vertical niche partitioning, contributes to the deep chlorophyll maximum, and shapes the evolution of antenna systems, photoreceptor complements, and regulatory gain control (Graham et al. 2009; Raven et al. 2012; Croce and van Amerongen 2014). In shallow, turbid, or highly mixed waters, fluctuating intensity and variable spectra favor broad-band sensing and strong photoprotection, whereas stable, spectrally narrow low-light environments favor high-efficiency light harvesting with tight redox control. These differences reflect recombination and differential tuning of conserved photoreceptive and signaling components.

### Diversification as modular recombination of conserved components

Across lineages, related regulatory problems are solved by modular components combined in different ways. Primary endosymbiosis provided plastids and cyanobacterial gene heritage that contributed precursors for bilin- and flavin-linked sensory systems; later secondary/tertiary endosymbioses redistributed and expanded photoreceptive diversity in stramenopiles, haptophytes, and dinoflagellates (representative lineages in Figure S1) (Bhattacharya et al. 2004; Keeling 2013; Archibald 2015; Dorrell and Bowler 2017). The result is not a single “optimal” photoreceptor set, but repeated recombination and differential expansion or retention of receptor families connected to shared downstream signaling hubs.

Accordingly, present-day genomes encode a diverse but structured toolkit—rhodopsins (behavior), cryptochromes (timing), phototropins (organelle movement and physiology), phytochrome-like systems (red and far-red sensing), and aureochromes as lineage-specific blue-light transcription factors (Takahashi et al. 2007; Coesel 2024). The distribution of these photoreceptor families across algal lineages is summarized in Table S1. Ecological specialization often reflects not only which receptors are present, but how strongly they are functionally coupled to chloroplast redox signaling and stress-response pathways. For example, deepwater red algae combine efficient blue–green harvesting with regulatory systems adapted to persistently low irradiance, whereas many green algae in shallow or terrestrial habitats emphasize photoprotection and broader spectral responsiveness (Raven et al. 2012; Raven and Giordano 2014).

**Figure 6:**
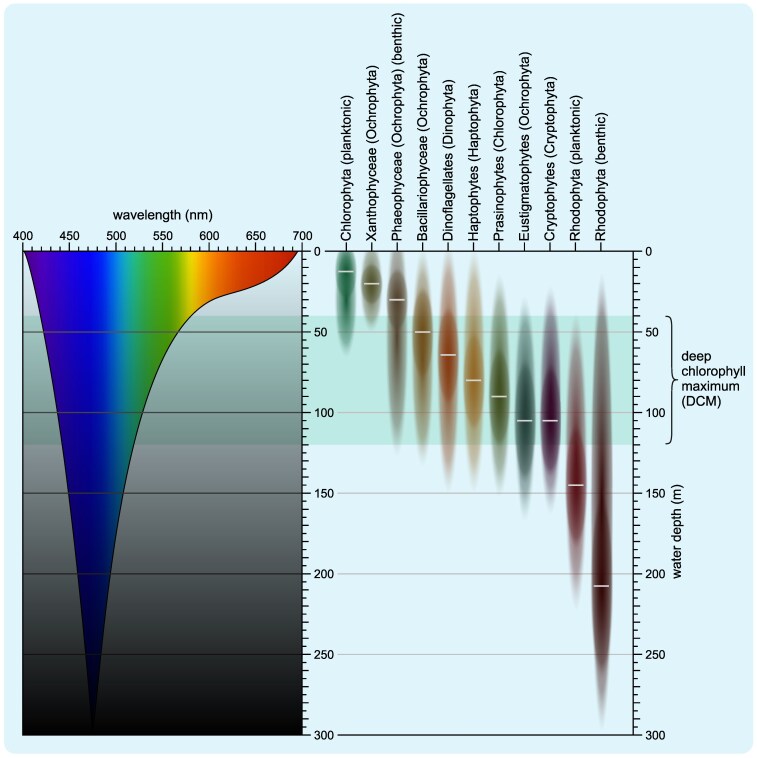
Spectral light attenuation and vertical niche partitioning among major eukaryotic algal lineages. The left panel illustrates the depth-dependent spectral composition of downwelling irradiance in clear oceanic waters, showing the progressive attenuation of longer wavelengths (red and orange) and the increasing dominance of blue light with depth (400 to 700 nm). The right panel depicts schematic vertical distribution ranges (semitransparent envelopes) and centers of maximum relative abundance (white bars) of major eukaryotic algal groups, arranged according to the depth of their abundance maxima. The shaded band indicates the deep chlorophyll maximum, where phytoplankton biomass and chlorophyll concentration commonly peak. Many pelagic phytoplankton groups—including diatoms, dinoflagellates, haptophytes, prasinophytes, eustigmatophytes, and cryptophytes—show substantial overlap within the DCM, whereas chlorophytes and xanthophytes tend to occupy shallower layers, and rhodophytes extend to the deepest photic-zone niches in both planktonic and benthic habitats. Conceptual illustration drawn after (Renema 2018) and supplemented with information presented in studies on underwater light attenuation, deep chlorophyll maxima, and vertical niche partitioning in algae (Cullen 1982; Falkowski and Raven 2007; Kirk 2010; Mignot et al. 2014; Larkum et al. 2020).

Behavioral and developmental modules coevolve with these architectures: Phototaxis supports navigation of steep vertical gradients in motile taxa, while benthic macroalgae tune morphology and pigment ratios to depth-dependent spectra, arising from wavelength-dependent attenuation of light in the water column (Fig. 6) (Cunningham et al. 2013; Renema 2018). Variation in cryptochrome- and aureochrome-centered signaling contributes to species-specific blue-light responses that facilitate niche partitioning among sympatric taxa (Mann et al. 2020; Im et al. 2024a). Together, these patterns illustrate how habitat-specific selection shapes lineage-specific signaling configurations through recombination and tuning of conserved components, linking molecular light sensing to organismal traits and ecological niche structure along environmental light gradients (Fig. 4).

### Ecological success across light environments

The same light-responsive networks that coordinate photoprotection, growth, timing, and reproduction also shape ecological performance across diverse light environments (Fig. 5). In turbulent waters, rapid adjustment of excitation pressure through NPQ, state transitions, and chloroplast/antenna regulation enhances persistence under fluctuating irradiance (Lavaud et al. 2007; Lavaud and Goss 2014; Taddei et al. 2018). In UV-rich polar/alpine or terrestrial microhabitats, protective pigmentation and stress-responsive switching enable survival under extreme irradiance variability (Remias et al. 2009, 2016; Leya 2013; Karsten and Holzinger 2014; Chevrollier et al. 2023; Healy and Khan 2023; Almela et al. 2025). In hypersaline and geothermal systems, modified carotenoid profiles and photosystem stability mechanisms illustrate how environmental stress reshapes light/redox regulation (Papageorgiou 2004; Haniewicz et al. 2018; Chantzistrountsiou et al. 2023). Other extreme habitats (eg endolithic or aerial environments) similarly select for desiccation tolerance and UV protection linked to light sensing (Friedmann 1982; Siebert et al. 1996; Sun 2013; Karsten et al. 2016; Medwed et al. 2021).

### Biogeochemical relevance

At ecosystem scale, these regulatory variations are consequential because photosynthetic algae collectively form much of the base of aquatic food webs and contribute approximately half of global net primary productivity (Field et al. 1998; Behrenfeld et al. 2005, 2016; Siegel et al. 2023; Xue et al. 2024). Light-tuned regulatory networks therefore influence carbon fixation and nutrient cycling by determining when and where phytoplankton sustain high photosynthetic performance under fluctuating irradiance and resource limitation (Behrenfeld et al. 2006; Falkowski and Raven 2007).

Algal diversification reflects habitat-driven rewiring of partly conserved and recurrent signaling modules: Environments filter spectra and variability, and lineages respond by adjusting sensor modules, coupling to chloroplast redox signals, and the gain/feedback properties of downstream networks—producing a mosaic of ecotypes built on a common regulatory logic (Duanmu et al. 2017; Petersen et al. 2021). Current work is moving toward predictive models, but a central unresolved challenge is to explain how heterogeneous inputs—spectral quality, intensity, temporal structure, and metabolic state—are compressed into a limited set of robust cellular decisions across highly variable environments. Such models will likely need to be developed in a stepwise and lineage-aware manner rather than as a single universal framework. A first level could focus on experimentally tractable single-species systems in which photoreceptor complement, cell geometry, motility or developmental state, and core physiological light responses are accessible. A second level could incorporate abiotic environmental structure, including spectral gradients, nutrient availability, temperature, mixing, and diel or seasonal light cycles. Only in a later step should population-level interactions, such as density effects, life-cycle transitions, grazing, symbiosis, or interspecific competition, be added. In this framework, generalization is most realistic at the level of recurring functional constraints, including spectral filtering, energetic–sensory coupling, second-messenger integration, and trade-offs between growth, protection, and movement. By contrast, the specific implementation of these principles is expected to remain lineage- and lifestyle-dependent, reflecting differences in photoreceptor repertoires, plastid architecture, cell size, motility, multicellularity, and habitat structure.

## Emerging concepts and future directions

Current work in algal photobiology shows that light sensing and light utilization are integrated through regulatory networks that couple photoreceptor inputs with chloroplast redox and metabolic signals (Rochaix 2011; Petroutsos et al. 2016). These networks connect complex optical environments with coordinated physiological, behavioral, and developmental responses. The diversity and evolutionary history of algal photoreceptors have recently been synthesized in detail (Hallmann 2025); here, the focus shifts to approaches that integrate these components into predictive, systems-level models. The main challenge is no longer simply to identify additional components, but to understand how sensory diversity, metabolic feedback, and network organization together generate robust responses across fluctuating light regimes and shape cellular decisions across scales.

### Genomics, structural prediction, and discovery of new light sensors

Expanded genomic and transcriptomic resources, including PhycoCosm and the One Thousand Plant Transcriptomes initiative, have accelerated discovery in algal photobiology (Carpenter et al. 2019; One Thousand Plant Transcriptomes Initiative 2019; Grigoriev et al. 2021). Comparative genomics increasingly reveals lineage-specific expansions, domain recombinations, and previously unrecognized photoreceptor candidates, including opsins, photolyases, bilin-binding proteins, and hybrid sensor–effector architectures.

AI-assisted structural prediction and modeling enable functional hypotheses prior to experimental characterization. Combining these predictions with heterologous expression, spectroscopy, and electrophysiology will uncover additional receptor classes with novel spectral properties and kinetics (Boyden et al. 2005; Spudich 2006). These approaches will expand our understanding of algal sensory biology and the repertoire of light-responsive modules for biotechnology and synthetic biology, many of which interface with shared intracellular signaling hubs that integrate light perception with metabolic state (Fig. 3).

### Toward systems-level models of integrated light signaling

In natural habitats, algal cells and populations often experience fluctuating light intensity, shifting spectral composition, and concurrent stresses such as nutrient limitation, temperature variation, or salinity change. Understanding this integration requires moving beyond single-pathway analyses to network-level approaches combining transcriptomics, proteomics, metabolomics, and high-resolution imaging (Fischer et al. 2007; Schmollinger et al. 2014).

Linking these datasets to mechanistic models should help clarify, first in selected systems, how photoreceptor signals, chloroplast redox states, and second-messenger dynamics interact to produce discrete, threshold-like outputs, such as division, phototaxis, or reproductive switching. An important goal is to identify the minimal recurring features that confer sensitivity, robustness, memory, and reversibility in fluctuating light environments and to determine whether these motifs recur across distantly related algal lineages. Placing these network organizations in an evolutionary context will clarify how algal lineages have been rewired to match ecological light regimes (Duanmu et al. 2017; Hallmann 2025). Combining laboratory perturbation studies with field-resolved optical and environmental datasets will allow tests of whether cellular regulatory architectures predict population dynamics, bloom behavior, and depth-dependent niche occupation in selected well-defined systems.

Overall, diverse algal systems provide useful models for studying how environmental light information is translated into biological function across scales, provided that such integration is developed stepwise and with explicit attention to lineage and lifestyle. Integrating genomics, structural biology, systems modeling, and ecological data may progressively support predictive models linking molecular light sensing to cellular regulation, ecological performance, and biogeochemical dynamics.

## Advances box

- **Integrated signaling framework**: Photoreceptor inputs converge with chloroplast redox and metabolic signals to coordinate physiology, development, and behavior across biological scales.
- **Evolutionary modularity**: Diverse algal photoreceptor repertoires reflect lineage-specific recombination of conserved sensory modules.
- **Multiscale regulation**: Light-dependent processes span scales from ion fluxes and transcriptional networks to organismal traits and ecological distributions.
- **Rapid expansion of genomic resources**: Large comparative datasets now enable systematic discovery and functional annotation/prediction of novel algal light-sensing proteins.
- **Technological impact**: Algal photoreceptors, particularly channelrhodopsins, have enabled major advances in optogenetics and synthetic biology.

## Outstanding questions box

- How are intensity, spectral quality, direction, and temporal light signals integrated into coherent cellular decisions?
- What is the full diversity of algal photoreceptors? Do additional sensor families remain undiscovered in poorly sampled lineages or extreme environments?
- How are chloroplast redox signals mechanistically and quantitatively coupled to photoreceptor signaling pathways?
- How does regulatory network architecture diversify across lineages and ecological niches, and what constraints shape this diversification?
- Can experimentally grounded predictive models link molecular light sensing to ecosystem-level processes such as primary productivity, bloom dynamics, and resilience under climate-driven change?
- What are the minimal design principles of light-sensing networks that generate robustness, plasticity, and evolvability across fluctuating environments?

## Supplementary Material

kiag524_Supplementary_Data

## Data Availability

No new primary data were generated for this study. All information discussed in this review is derived from previously published studies cited in the reference list.
