# Supplementary material for: Light-dependent processes in algae across scales: from cellular physiology to ecological organization
Source: Plant Physiol. 2026 Jul 24;201(4):kiag524. doi: 10.1093/plphys/kiag524 (PMC13436692; doi:10.1093/plphys/kiag524)
Supplement: Supplementary file 1 [file kiag524_supplementary_data.zip › Supplementary Figure S1.pdf]

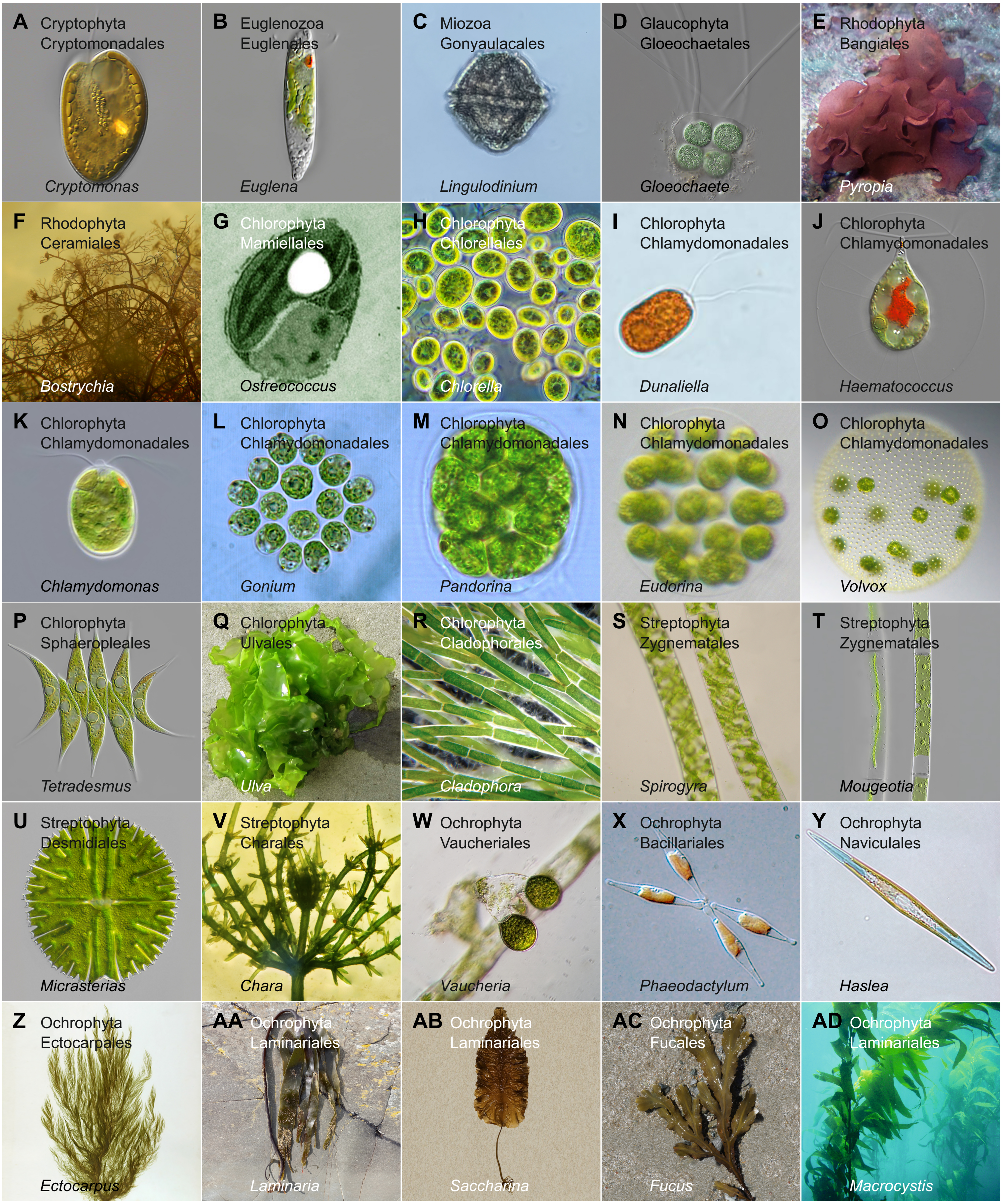

Supplementary Figure S1. Representative diversity of eukaryotic algae discussed in the text. Panels A–AD provide visual references for representative taxa from major eukaryotic algal lineages. Secondary plastid–bearing lineages include Cryptophyta (A), Euglenozoa (B), and Miozoa/Dinophyceae (C), which represent independent acquisitions of secondary plastids and therefore do not form a monophyletic photosynthetic assemblage, as well as Ochrophyta (Stramenopiles) (W–AD), which share a red algal–derived secondary plastid and a conserved plastid protein-import machinery. Archaeplastida (D–V) are shown from basal lineages (Glaucophyta, Rhodophyta) to Chlorophyta and

Streptophyta, highlighting major transitions in cellular organization. Within Ochrophyta, brown algae (Z–AD) illustrate increasing morphological and life-cycle complexity within Phaeophyceae, ranging from filamentous forms to large kelps. Taxonomic information (phylum, order, genus) is indicated within the panels. Images were compiled from multiple sources, including Creative Commons–licensed material, material reproduced with permission, and original images generated by the author. Sources and licenses of images are provided below.

Sources and licenses of images:

Panels	Taxon / Image Title	Author / Photographer	Original Source (Platform)	Direct URL	License	License URL
**A**	*Cryptomonas erosa*	Martin Kreutz	Real Micro Life	https://realmicrolife.com/cryptomonas-erosa/	Permission obtained from the copyright holder	
**B**	*Euglena agilis*	Martin Kreutz	Real Micro Life	https://realmicrolife.com/euglena-agilis/	Permission obtained from the copyright holder	
**C**	*Lingulodinium polyedra*	Klisarova et al. (2025)	Klisarova et al. (2025). Diversity-Basel 17, 105.	https://doi.org/10.3390/d17020105	CC BY 4.0	https://creativecommons.org/licenses/by/4.0/
**D**	*Gloeochaete wittrockiana*	Martin Kreutz	Real Micro Life	https://realmicrolife.com/gloeochaete-wittrockiana/	Permission obtained from the copyright holder	
**E**	*Pyropia* sp.	Armin Hallmann			Created by the author	
**F**	*Bostrychia scorpioides*	Duarte Frade	iNaturalist	https://www.inaturalist.org/photos/33878068	CC BY 4.0	https://creativecommons.org/licenses/by/4.0/
**G**	*Ostreococcus lucimarinus*	Hervé Moreau	Microbe Wiki	https://microbewiki.kenyon.edu/index.php/File:Ostta.jpg	Permission obtained from the copyright holder	
**H**	*Chlorella* sp.	Andrei Savitsky	Wikimedia Commons	https://commons.wikimedia.org/wiki/File:%D0%9E%D0%B4%D0%BD%D0%BE%D0%BA%D0%BB%D0%B5%D1%82%D0%BE%D1%87%D0%BD%D1%8B%D0%B5_%D0%B2%D0%BE%D0%B4%D0%BE%D1%80%D0%BE%D1%81%D0%BB%D0%B8_%D1%85%D0%BB%D0%BE%D1%80%D0%B5%D0%BB%D0%BB%D0%B0.jpg	CC BY 4.0	https://creativecommons.org/licenses/by/4.0/
**I**	*Dunaliella salina*	Barmshuri et al. (2022)	Barmshuri et al. (2022). bioRxiv, 2022.2012.2013.520199.	https://doi.org/10.1101/2022.12.13.520199	CC0 1.0	https://creativecommons.org/publicdomain/zero/1.0/
**J**	*Haematococcus pluvialis*	Martin Kreutz	Real Micro Life	https://realmicrolife.com/haematococcus-lacustris/	Permission obtained from the copyright holder	
**K**	*Chlamydomonas reinhardtii*	Armin Hallmann			Created by the author	
**L**	*Gonium pectorale*	Armin Hallmann			Created by the author	
**M**	*Pandorina morum*	Armin Hallmann			Created by the author	
**N**	*Eudorina* sp.	Armin Hallmann			Created by the author	
**O**	*Volvox carteri*	Armin Hallmann			Created by the author	
**P**	*Tetradesmus lagerheimii*	Martin Kreutz	Real Micro Life	https://realmicrolife.com/tetradesmus-lagerheimii/	Permission obtained from the copyright holder	
**Q**	*Ulva lactuca*	Kyle Van Houtan	iNaturalist	https://www.inaturalist.org/photos/102370	CC BY 4.0	https://creativecommons.org/licenses/by/4.0/
**R**	*Cladophora* sp.	Olivier Dugornay	Wikimedia Commons	https://commons.wikimedia.org/wiki/File:Algue_verte_(Cladophora)_(Ifremer_00656-76791).jpg	CC BY 4.0	https://creativecommons.org/licenses/by/4.0/
**S**	*Spirogyra* sp.	Luka S. (someplant)	iNaturalist	https://www.inaturalist.org/observations/178227504	CC0 1.0	https://creativecommons.org/publicdomain/zero/1.0/
**T**	*Mougeotia scalaris*	Martin Kreutz	Real Micro Life	https://realmicrolife.com/mougeotia-scalaris/	Permission obtained from the copyright holder	
**U**	*Micrasterias rotata*	Martin Kreutz	Real Micro Life	https://realmicrolife.com/micrasterias-rotata/	Permission obtained from the copyright holder	
**V**	*Chara globata*	Nurashov et al. (2023)	Nurashov et al. (2023). Plants (Basel) 12, 368.	https://doi.org/10.3390/plants12020368	CC BY 4.0	https://creativecommons.org/licenses/by/4.0/
**W**	*Vaucheria canalicularis*	David Bird	iNaturalist	https://www.inaturalist.org/photos/266623770	CC0 1.0	https://creativecommons.org/publicdomain/zero/1.0/
**X**	*Phaeodactylum tricornutum*	Alessandra de Martino and Chris Bowler	Wikipedia	https://en.wikipedia.org/wiki/Phaeodactylum#/media/File:Phaeodactylum_tricornutum.png	CC BY 2.5	https://creativecommons.org/licenses/by/2.5/
**Y**	*Haslea ostrearia*	Gabed et al. (2022)	Gabed et al. (2022). Mar Drugs 20, 234.	https://doi.org/10.3390/md20040234	CC BY 4.0	https://creativecommons.org/licenses/by/4.0/
**Z**	*Ectocarpus longifructus*	Pierre-Louis Crouan and Hippolyte-Marie Crouan	Wikimedia Commons	https://commons.wikimedia.org/wiki/File:Ectocarpus_longifructus_Crouan.jpg	PDM 1.0	https://creativecommons.org/publicdomain/mark/1.0/
**AA**	*Laminaria digitata*	sugarsnap_t	iNaturalist	https://www.inaturalist.org/observations/179353584	CC BY 4.0	https://creativecommons.org/licenses/by/4.0/
**AB**	*Saccharina latissima*	Arlov et al. (2024)	Arlov et al. (2024). Algal Research 78, 103421.	https://doi.org/10.1016/j.algal.2024.103421	CC BY 4.0	https://creativecommons.org/licenses/by/4.0/
**AC**	*Fucus serratus*	Stephen James McWilliam	iNaturalist	https://uk.inaturalist.org/observations/90636695	CC0 1.0	https://creativecommons.org/publicdomain/zero/1.0/
**AD**	*Macrocystis* sp.	Claire Fackler	Wikipedia	https://de.wikipedia.org/wiki/Datei:Sanc0063_-_Flickr_-_NOAA_Photo_Library.jpg	CC BY 2.0	https://creativecommons.org/licenses/by/2.0/
