# Supplementary material for: Light-dependent processes in algae across scales: from cellular physiology to ecological organization
Source: Plant Physiol. 2026 Jul 24;201(4):kiag524. doi: 10.1093/plphys/kiag524 (PMC13436692; doi:10.1093/plphys/kiag524)
Supplement: Supplementary file 1 [file kiag524_supplementary_data.zip › Supplementary Figure S2.pdf]

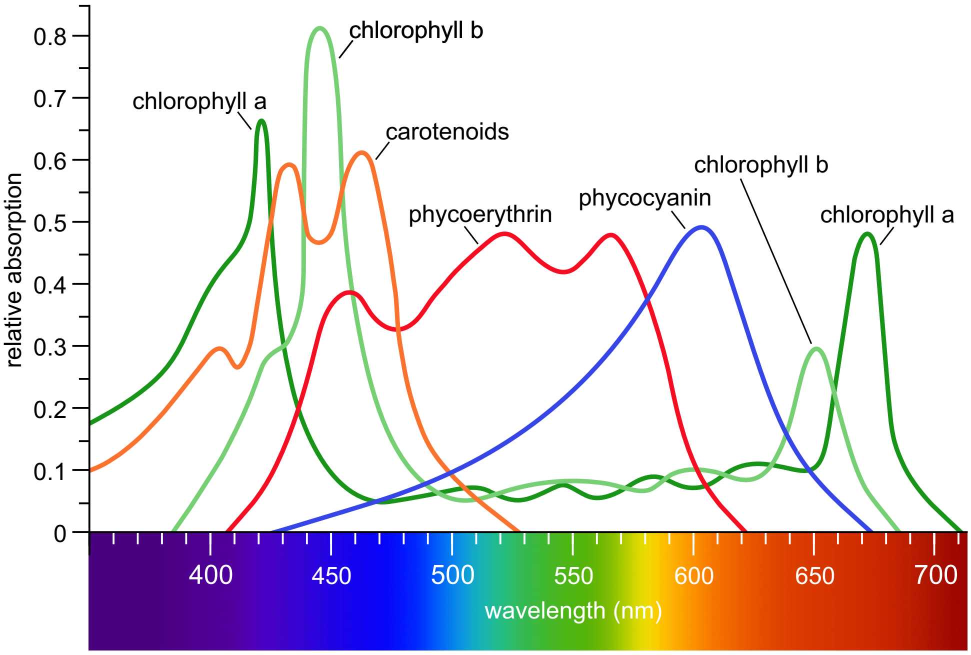

Supplementary Figure S2. Spectral absorption properties of major algal light-harvesting pigments. Absorption spectra of key photosynthetic pigments, such as chlorophyll a, chlorophyll b, carotenoids, phycoerythrin, and phycocyanin, showing how different pigment systems capture distinct regions of the visible light spectrum. The spectra were compiled from published datasets (Kobayashi et al., 2013; Geuer et al., 2023; Ángeles et al., 2025) and redrawn for clarity.
