# Supplementary material for: Light-dependent processes in algae across scales: from cellular physiology to ecological organization
Source: Plant Physiol. 2026 Jul 24;201(4):kiag524. doi: 10.1093/plphys/kiag524 (PMC13436692; doi:10.1093/plphys/kiag524)
Supplement: Supplementary file 1 [file kiag524_supplementary_data.zip › Supplementary Table S1.pdf]

Supplementary Table S1. Comparative distribution of major photoreceptor classes across algal lineages

Legend:

●	well established and functionally characterized
◐	present (supported by genomic evidence and/or limited functional data)
○	rare, lineage-specific, or poorly characterized
—	not reported

**Photoreceptor class**	**Representative taxa / examples**	**Chlorophyta**	**Rhodophyta**	**Stramenopiles**	**Dinoflagellata**	**Cryptophyta**	**Dominant functional roles**
phototropins (LOV-domain kinases)	*Chlamydomonas, Volvox*	●	○	—	◐	◐	phototaxis, chloroplast movement, NPQ, regulation of carbon metabolism
aureochromes (LOV–bZIP TFs)	*Phaeodactylum, Vaucheria*	—	—	●	—	—	blue-light–regulated transcription, development, circadian entrainment
cryptochromes	*Chlamydomonas, Ostreococcus*	●	◐	●	○	◐	circadian rhythms, developmental transitions, transcriptional control
BLUF-domain proteins	*Euglena, Chlamydomonas*	◐	○	●	○	◐	photobehavior, photoacclimation, metabolic regulation
rhodopsins (type-1 microbial)	*Chlamydomonas, Lingulodinium*	●	○	◐	●	●	phototaxis, photophobic responses, ion transport, cNMP signaling
phytochromes	*Phaeodactylum, Pyropia*	◐	◐	●	○	◐	red/far-red light sensing, spectral acclimation, regulation of growth and life-cycle transitions
neochromes (phytochrome–LOV hybrids)	*Mougeotia*	◐	—	—	—	—	integration of blue and red light signals, phototropism
UV-B photoreceptors (UVR8)	*Chlamydomonas*	●	—	○	—	—	UV-B stress signaling, photoprotection, acclimation to UV radiation

Note: Presence indicates detection in representative genomes and/or functional studies; absence reflects lack of current evidence rather than definitive loss.
